# Association between maternal anxiety/depression in pregnancy and the development of offspring eczema/AD: a meta-analysis based on cohort studies

**DOI:** 10.3389/fped.2025.1734662

**Published:** 2026-01-13

**Authors:** Mengjiao Yu, Qiufeng Zhang, Junyi Chen, Jingyu Yang, Zhechuan Bai, Junjie Wang

**Affiliations:** 1 Emergency Medical Center, Ningbo Hospital of Integrated Traditional Chinese and Western Medicine, Ningbo, Zhejiang, China; 2 The Second School of Clinical Medicine, Zhejiang Chinese Medical University, Hangzhou, Zhejiang, China; 3 The School of Nurse, Zhejiang Chinese Medical University, Hangzhou, Zhejiang, China

**Keywords:** anxiety, atopic dermatitis, depression, eczema, maternal, offspring

## Abstract

**Background:**

This meta-analysis investigates the association between maternal anxiety/depression during pregnancy and the development of eczema/atopic dermatitis (AD) in offspring.

**Methods:**

A literature search was conducted across four electronic databases (PubMed, Web of Science, Embase, and the Cochrane Library) for studies published from database inception until July 2025. In this study, maternal depression and anxiety were defined as conditions physician-diagnosed or assessed with standardized scales during pregnancy. The primary outcome was the incidence of eczema/AD in the offspring.

**Result:**

A total of 12 cohort studies were included in this meta-analysis. Pooled results indicated that maternal depression [odds ratio (OR) = 1.06, 95% confidence interval (95% CI) = 1.01–1.11, *p* = 0.015] or anxiety (OR = 1.11, 95% CI = 1.03–1.19, *p* = 0.005) during pregnancy was potentially associated with a higher incidence of offspring eczema and AD. Subgroup analysis revealed that there was a higher incidence of AD in offspring with maternal anxiety during pregnancy (OR = 1.24, *p* = 0.028), while no significant difference was observed in the incidence of eczema (*p* = 0.286). A higher incidence of offspring eczema/AD was observed in offspring of both Eastern (OR = 1.13, *p* = 0.043) and Western (OR = 1.34, *p* = 0.049) countries. Moreover, the incidence was higher in offspring when maternal anxiety was identified in the first (OR = 1.13, *p* = 0.036) or second (OR = 1.25, *p* = 0.010) trimester, whereas no significant difference was found for exposure in the third trimester (*p* = 0.152). For maternal depression during pregnancy, offspring had a higher incidence of AD (OR = 1.17, *p* < 0.001), while no significant difference was observed for eczema (*p* = 0.145). Furthermore, the incidence of offspring eczema/AD was higher in Eastern countries (OR = 1.14, *p* = 0.035), while Western countries group showed no significant difference (*p* = 0.111). Additionally, when analyzed by timing of exposure, the incidence was higher when depression was identified in the second trimester (OR = 1.30, *p* = 0.027), with no significant difference found in the third trimester (*p* = 0.163).

**Conclusion:**

This study suggests that maternal depression/anxiety during pregnancy is potentially associated with the development of eczema/AD in offspring.

## Introduction

Eczema is a dermatological condition characterized by erythema, swelling, desquamation, and xerosis. Among the various subtypes of eczema, atopic dermatitis (AD) is the most prevalent (1). This chronic inflammatory skin disorder is marked by pruritus, erythema, edema, and exudative lesions. Furthermore, AD affects up to 25% of children under 7 years old, making it one of the most common chronic disorders (2). Current research has linked several prenatal exposures, such as maternal psychosocial stress, passive smoking, dietary patterns, and alcohol consumption, to the development of eczema/AD in offspring (3–6). In particular, some studies indicate that maternal anxiety/depression during pregnancy may be associated with an increased incidence of eczema/AD in children (3, 7). However, a definitive academic consensus on this issue remains elusive (8). Therefore, this study aims to resolve this controversy by reviewing cohort studies to investigate the association between maternal anxiety/ depression during pregnancy and the subsequent development of eczema/AD in offspring.

## Method

The primary objective of this meta-analysis is to investigate the relationship between maternal anxiety/depression during pregnancy and offspring suffering from eczema/AD. This study conducted systematic searches in PubMed, Embase, Web of Science, and Cochrane Library from database inception to July 2025 utilizing the following terms: [(offspring OR newborn OR neonate OR infant OR baby OR progeny OR children OR child OR kids OR toddler OR adolescent OR teenager) AND (dermatitis, atopic OR eczema OR atopic dermatitis OR atopic eczema)] AND [(pregnant OR pregnancy OR pregnant woman OR antenatal OR prenatal OR postpartum OR gestation OR perinatal OR pregnancy-related OR maternal) AND (depressive OR depression OR depressive state OR anxiety OR anxious)]. Additionally, to ensure comprehensiveness of searches, the references of all included articles would be also screened for additional eligible studies. A detailed description of the search strategy is available in the Supplementary Search Strategy. This study adhered to the Preferred Reporting Items for Systematic Reviews and Meta-Analyses guidelines (PRISMA) guidelines with registration on PROSPERO (CRD420251113756).

### Inclusion and exclusion criteria

Inclusion and exclusion criteria for this study were defined according to the PICOS (Population, Intervention, Comparison, Outcomes, Study design) framework:

P: children;
I: maternal exposure to anxiety/depression during pregnancy;
C: absence of maternal exposure to anxiety/depression during pregnancy;
O: occurrence of eczema/AD in children;
S: cohort studies.

Exclusion criteria also comprised non-English studies, studies with unavailable full texts, and articles containing data not amenable to statistical analysis. When the study had been updated, only the most comprehensive or recent version was retained for inclusion.

### Data extraction

Maternal anxiety/depression exposure was defined in two ways: (1) a clinical diagnosis based on the International Classification of Diseases or the Diagnostic and Statistical Manual of Mental Disorders, Fourth Edition or Fifth Edition; or (2) symptom assessment using self-reported scales such as the Edinburgh Postnatal Depression Scale (EPDS) or the State-Trait Anxiety Inventory (STAI) (9). The exposure window covered the entire pregnancy. For the division of pregnancy periods, we adopted the definitions explicitly provided in the original studies whenever available. If not specified, the following international standard trimesters were applied: first trimester (weeks 1–12), second trimester (weeks 13–27), and third trimester (week 28 until delivery) (10). The occurrence of eczema/AD was identified through clinical diagnosis or parental report.

Two reviewers independently performed data abstraction from included studies. The following data were extracted: authors, publication year, country, study design, participant characteristics (numbers, exposure factors, evaluation tools), covariates, outcome assessment methods, prevalence of eczema/AD. Discrepancies between independent reviewers were adjudicated through consultation with a third reviewer.

### Assessment of quality

Quality of eligible cohort studies underwent appraisal via the Newcastle-Ottawa Scale, evaluating three critical domains: Selection, Comparability, and Outcomes. Studies attaining scores≥6 were classified as high-quality. This evaluation was independently performed by two reviewers, with discrepancies adjudicated through consensus discussion involving a third reviewers.

### Statistical analysis

Statistical analysis was performed using Stata 12.0. The relationship between maternal anxiety/depression during pregnancy and the offspring suffering from eczema/AD was examined through pooled odds ratio (OR) with corresponding 95% confidence interval (95% CI). An OR >1 indicated a positive association between maternal depression/anxiety during pregnancy and offspring suffering from eczema/AD, whereas an OR <1 demonstrated an inverse association. Given the heterogeneity across studies in terms of race, diagnostic criteria for eczema/AD, education, and assessment tools for depression/anxiety, a random-effects model was applied to enhance the reliability of this study. Heterogeneity levels were evaluated employing Cochrane's *Q*-test alongside *I*^2^ statistics with predefined interpretation criteria where *I*^2^ below 25% represented low heterogeneity, values between 25% and 50% denoted moderate heterogeneity, and estimates above 50% reflected high heterogeneity. Sensitivity analysis was conducted to assess result stability, and publication bias was examined using Begg's test. A two-sided test was implemented for all analyses, with significance determined when *p* < 0.05.

## Result

### Studies selection

**Figure 1:**
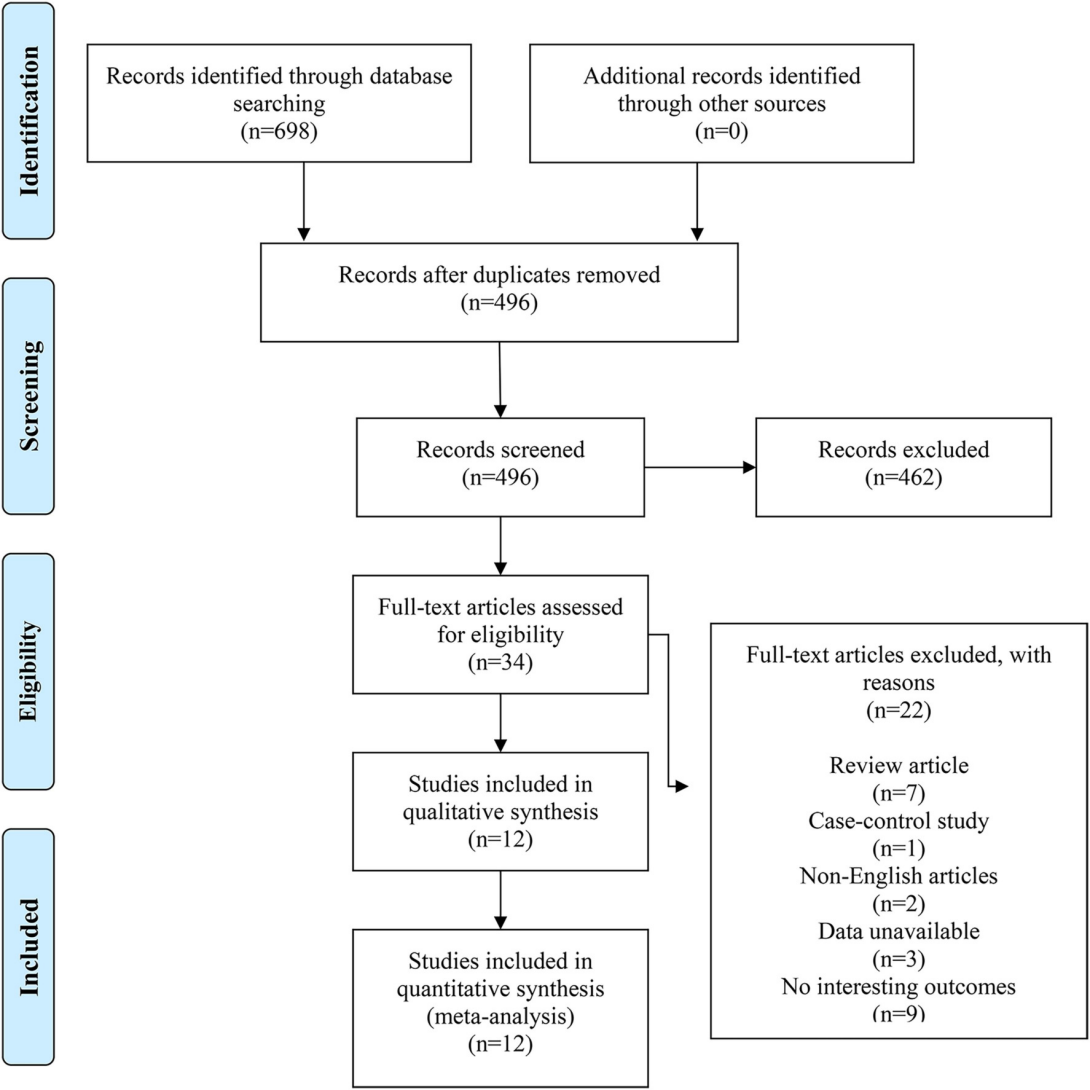
Flow diagram of the selection process.

The comprehensive search strategy across four electronic databases initially yielded 698 records. Following duplicate removal, 496 unique articles remained. Two reviewers independently evaluated these records based on titles and abstracts, excluding 462 irrelevant articles and retaining 34 for full-text assessment. After exclusions comprising 7 review articles, 1 case-control study, 2 non-English publications, 3 articles with unavailable data, and 9 articles reporting no interesting outcomes, 12 studies qualified for meta-analytic synthesis and were incorporated into the meta-analysis (11–22). The complete selection workflow is detailed in Figure 1.

### Study characteristics

Study characteristics are detailed in Table 1 and Supplementary Table S1. This study incorporated 12 cohort studies which were published between 2015 and 2025. Geographically, the studies encompassed both Eastern and Western countries (Singapore (*n* = 2), China (*n* = 3), Korea (*n* = 1), France (*n* = 1), Finland (*n* = 1), Netherlands (*n* = 1), Canada (*n* = 2), Germany (*n* = 1)). Key covariates adjusted for included child's sex, maternal age, maternal education level, household income, family history of atopy, and gestational age. The exposure windows varied across studies, with most focusing on the second and third trimesters, while a minority focused on the first trimester. Outcome assessment relied predominantly on parental reports or physician diagnosis of eczema/AD.

**Table 1:** Characteristics of included cohort studies in the meta-analysis.

Author, year	Country	Exposure factors	No. of participants	Assessment tools	Outcome
Cheng, T. S. 2015	Singapore	Anxiety, depression	908	STAI, EPDS	Eczema
Zhou, C. 2017	France	Depression	1,039	CES-D	Eczema
Wei, D. 2020	China	Depression	8,580	Self-Rating Depression Scale	Eczema
Puosi, E. 2022	Finland	Anxiety, depression	1,305	Symptom Checklist-90, anxiety scale, EPDS	Eczema
Lau, H. X. 2022	Singapore	Anxiety, depression	261	STAI, EPDS	Eczema
Zhou, J. 2024	China	Anxiety	3,160	Chinese version of the Pregnancy-Related Anxiety Questionnaire	Eczema
Freeman, M. 2024	Canada	Anxiety, depression	1,968	Spielberger State Anxiety Inventory, EPDS	Eczema
Elbert, N. J. 2017	Netherlands	Anxiety, depression	5,205	Brief Symptom Inventory	Eczema
Wu, J. Y. 2025	China	Anxiety, depression	5,263	Generalized Anxiety Disorder Questionnaire, Patient Health Questionnaire	Atopic dermatitis
Chang, H. Y. 2016	Korea	Anxiety, depression	775	State-Trait Anxiety Inventory Trait subscale, CES-D	Atopic dermatitis
Letourneau, N. L. 2017	Canada	Anxiety	242	Pregnancy-Specific Anxiety Scale	Atopic dermatitis
Braig, S. 2017	Germany	Anxiety, depression	787	Hospital Anxiety and Depression Scale	Atopic dermatitis

CES-D, Centre for Epidemiologic Studies-Depression scale; EPDS, Edinburgh Postnatal Depression Scale; No., number; STAI, State Trait Anxiety Inventory.

Ten studies investigated maternal anxiety exposure during pregnancy, involving approximately 20,000 participants. Primary assessment tools included the STAI, the Symptom Checklist-90 anxiety scale, and the Chinese version of the Pregnancy-Related Anxiety Questionnaire. These studies examined anxiety symptom rather than clinically diagnosed anxiety disorder. The outcomes comprised eczema, reported in six studies, and AD, examined in four studies.

Ten studies, involving over 25,000 participants, investigated the association between depression exposure during pregnancy and development of offspring eczema/AD. Maternal depression was predominantly assessed using the EPDS and the Centre for Epidemiologic Studies Depression Scale. These studies examined depression symptom rather than clinically diagnosed depression disorder. Among the outcomes, eczema was reported in 7 studies and AD in 3 studies.

### Quality assessment

Quality assessment of the 12 included cohort studies was performed using the Newcastle-Ottawa Scale. Seven studies were rated 8 points while five studies were rated 7 points. All studies were considered high-quality. See Supplementary Table S2 for details.

### Analysis of the anxiety and eczema/AD relationship

**Figure 2:**
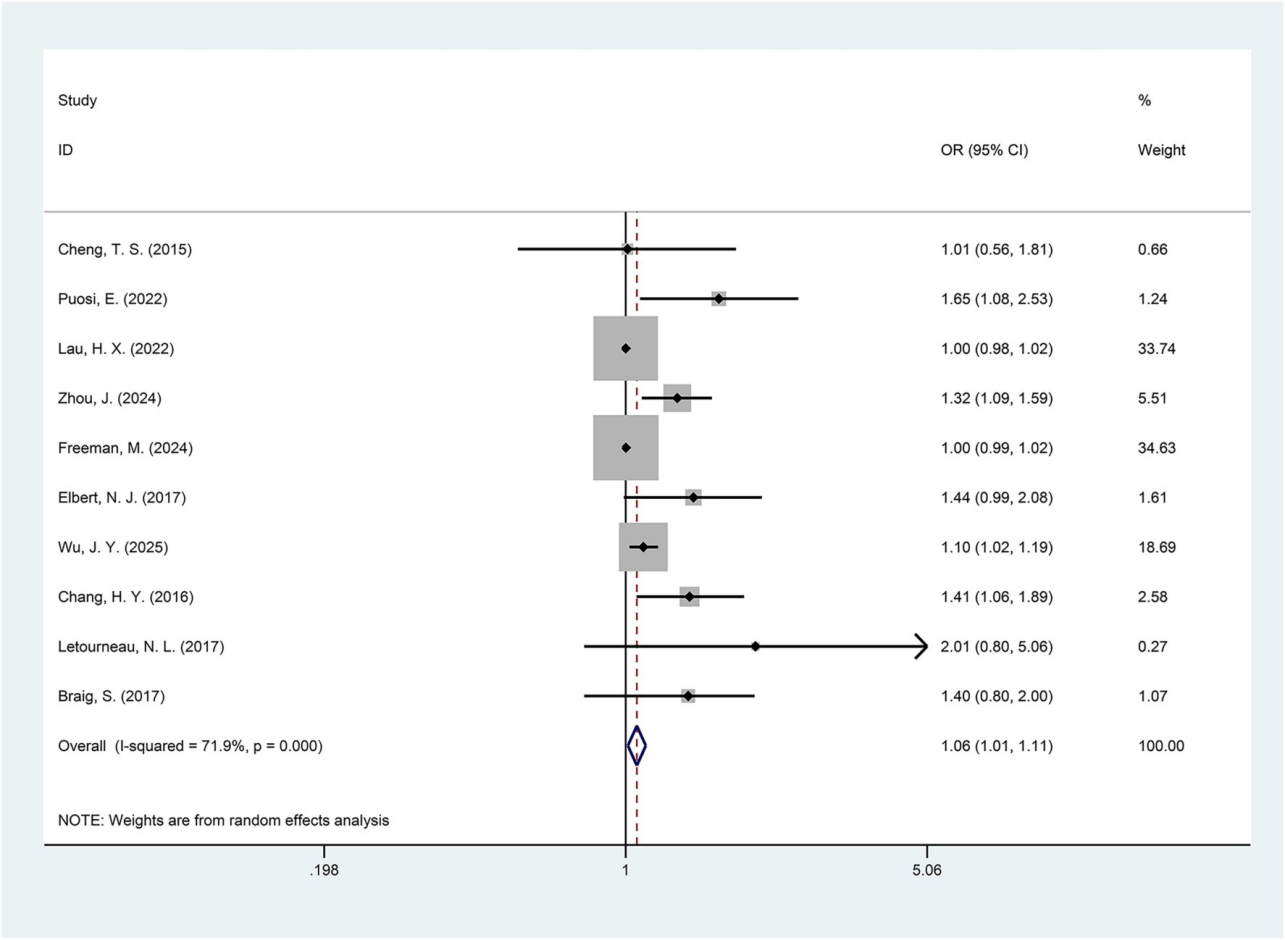
Forest plot of the relationship between maternal anxiety and the risk of offspring eczema/AD (*p* = 0.015).

An elevated prevalence of offspring suffering from eczema/AD was observed among mothers with anxiety during pregnancy when compared to the control group, as indicated by pooled effect sizes (OR = 1.06, 95% CI = 1.01–1.11, *p* = 0.015, *I*^2^ = 71.9%, Figure 2). However, the high level of heterogeneity suggested substantial variation among the included studies. This study also included various subgroup analyses. Subgroup analyses for anxiety dimensions revealed that neither maternal state anxiety, as measured by the STAI-S (OR = 1.00, 95% CI = 0.99–1.01, *p* = 0.916), nor trait anxiety (OR = 1.11, 95% CI = 0.86–1.44, *p* = 0.414) during pregnancy demonstrated a significant difference in the prevalence of offspring eczema or AD compared to the control group. Furthermore, subgroup analysis stratified by disease type indicated that offspring in the exposed group had a significantly increased prevalence of AD (OR = 1.24, 95% CI = 1.02–1.51, *p* = 0.028) compared to the control group, whereas the prevalence of eczema did not differ significantly between the exposed and control groups (OR = 1.02, 95% CI = 0.98–1.07, *p* = 0.296). Subgroup analysis stratified by region indicated a higher prevalence of eczema/AD in offspring of mothers with anxiety during pregnancy in both Eastern (OR = 1.13, 95% CI = 1.00–1.26, *p* = 0.043) and Western (OR = 1.34, 95% CI = 1.00–1.78, *p* = 0.049) populations compared to the control group. Stratification by the timing of exposure showed that the prevalence of eczema/AD was significantly higher in the offspring of mothers whose anxiety was detected in the first trimester (OR = 1.13, 95% CI = 1.01–1.27, *p* = 0.036) and the second trimester (OR = 1.25, 95% CI = 1.05–1.48, *p* = 0.010) compared to the control group. However, the group exposed to maternal anxiety identified in the third trimester showed no significant difference compared to the control group (OR = 1.19, 95% CI = 0.94–1.51, *p* = 0.152). Moreover, in studies adjusting for a family history of allergic diseases, the subgroup analysis revealed that offspring in the exposed group had an increased prevalence of eczema/AD compared to the control group (OR = 1.17, 95% CI = 1.04–1.33, *p* = 0.009). Subgroup analysis by assessment method demonstrated a higher prevalence in the exposed group with physician-diagnosed eczema/AD compared to the control group (OR = 1.40, 95% CI = 1.12–1.75, *p* = 0.003). However, there was no significant difference in the prevalence of parent-reported eczema/AD between the groups (OR = 1.04, 95% CI = 1.00–1.08, *p* = 0.077). Details of these subgroup analyses are presented in Table 2.

**Table 2:** Subgroup analysis of the association between maternal anxiety and the development of offspring eczema/AD.

Subgroups	No. of studies	OR	95% CI	*p*	*I*^2^ (%)
Anxiety dimensions					
STAI state	3	1.00	[0.99, 1.01]	0.916	0
STAI trait	3	1.11	[0.86, 1.44]	0.414	65.0
Disease types					
Eczema	6	1.02	[0.98, 1.07]	0.286	70.9
AD	4	1.24	[1.02, 1.51]	0.028	40.7
Region					
Eastern countries	5	1.13	[1.00, 1.26]	0.043	78.4
Western countries	5	1.34	[1.00, 1.78]	0.049	69.7
Timing of exposure					
First trimester	2	1.13	[1.01, 1.27]	0.036	23.9
Second trimester	3	1.25	[1.05, 1.48]	0.010	0
Third trimester	3	1.19	[0.94, 1.51]	0.152	82.9
Adjusted factors					
Family history of allergy adjusted	8	1.17	[1.04, 1.33]	0.009	70.2
Eczema/atopic dermatitis assessment method					
Physician-diagnosed	3	1.40	[1.12, 1.75]	0.003	0
Parent-reported	7	1.04	[1.00, 1.08]	0.077	72.2

95% CI, 95% Confidence intervals; AD, Atopic dermatitis; No., Number; OR, Odds ratio; STAI, State Trait Anxiety Inventory.

### Analysis of the depression and eczema/AD relationship

**Figure 3:**
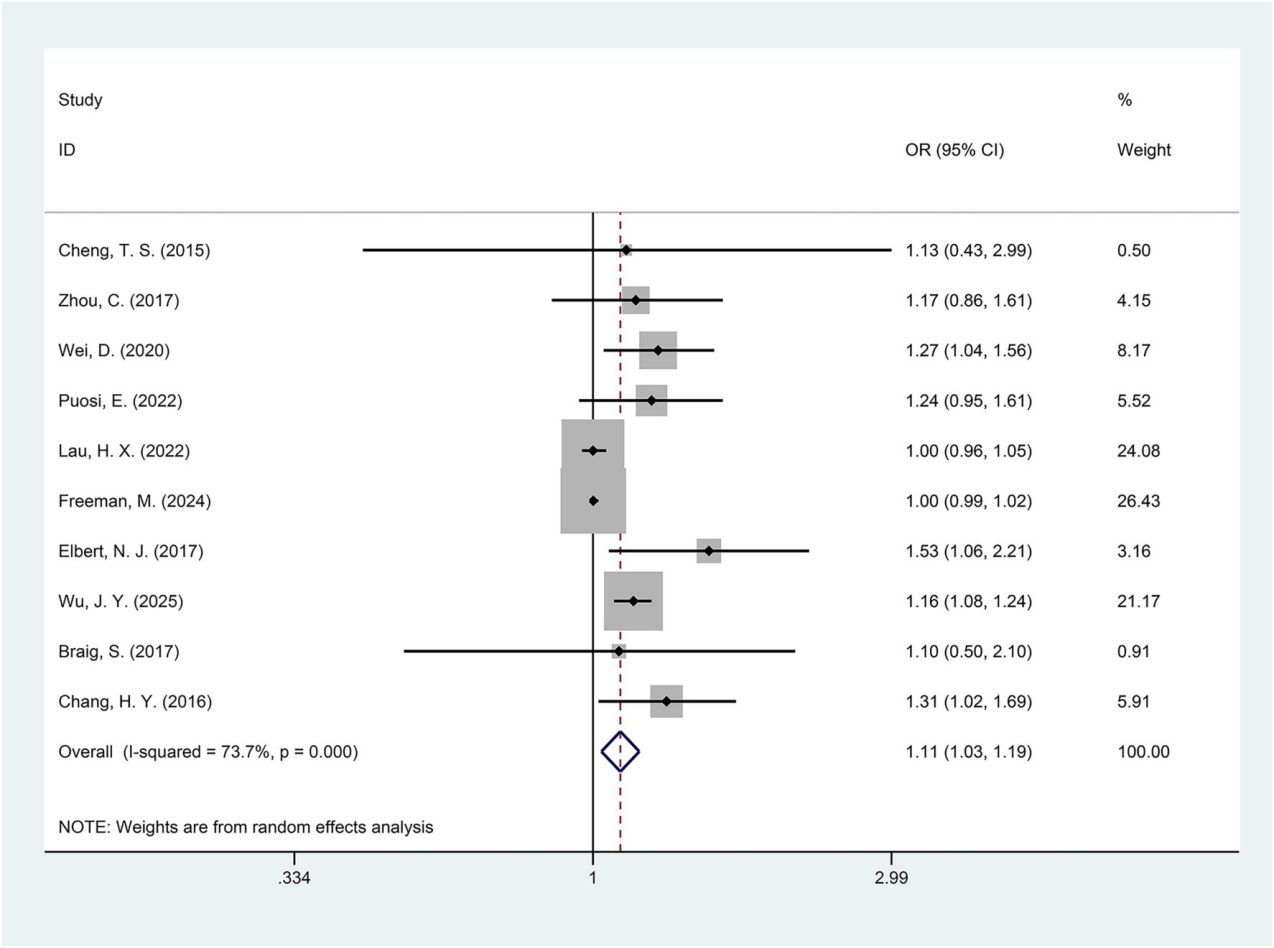
Forest plot of the relationship between maternal depression and the risk of offspring eczema/AD (*p* = 0.005).

The combined effect sizes indicated a higher prevalence of eczema/AD in offspring of mothers with depression during pregnancy compared to the control group (OR = 1.11, 95% CI = 1.03–1.19, *p* = 0.005, *I*^2^ = 73.7%, Figure 3). However, there was considerable heterogeneity among the studies included. Subgroup analysis by disease types revealed a greater prevalence of AD in the exposed offspring compared to the control group (OR = 1.17, 95% CI = 1.09–1.25, *p* < 0.001), while no significant difference in eczema prevalence was noted between the exposed and control groups (OR = 1.05, 95% CI = 0.98–1.11, *p* = 0.145). Analysis by region showed that offspring exposed to maternal depression during pregnancy in Eastern countries had a higher prevalence of eczema/AD compared to the control group (OR = 1.14, 95% CI = 1.01–1.29, *p* = 0.035), whereas no statistically significant difference was observed in Western countries (OR = 1.15, 95% CI = 0.97–1.36, *p* = 0.111). Maternal depression identified during the second trimester significantly increased the prevalence of eczema/AD in offspring compared to the control group (OR = 1.30, 95% CI = 1.03–1.64, *p* = 0.027). In contrast, no significant difference was observed between the exposed and control groups when depression was detected during the third trimester (OR = 1.16, 95% CI = 0.94–1.42, *p* = 0.163). In studies that adjusted for family history of allergic diseases, subgroup analysis revealed a higher prevalence of eczema/AD in the exposed offspring relative to the control group (OR = 1.17, 95% CI = 1.06–1.30, *p* = 0.003). The prevalence of eczema/AD was greater in the exposed offspring than in the control group, regardless of whether the diagnosis was made by a physician (OR = 1.25, 95% CI = 1.07–1.40, *p* = 0.006) or reported by parents (OR = 1.08, 95% CI = 1.00–1.16, *p* = 0.042). Moreover, maternal depression showed a stronger association with the development of physician-diagnosed eczema/AD in offspring than on parent-reported cases. Detailed information on the subgroup analysis can be found in Table 3.

**Table 3:** Subgroup analysis of the association between maternal depression and the development of offspring eczema/AD.

Subgroups	No. of studies	OR	95% CI	*p*	*I*^2^ (%)
Disease types					
Eczema	7	1.05	[0.98, 1.11]	0.145	56.6
Atopic dermatitis	3	1.17	[1.09, 1.25]	<0.001	0
Region					
Eastern countries	5	1.14	[1.01, 1.29]	0.035	78.7
Western countries	5	1.15	[0.97, 1.36]	0.111	53.6
Timing of exposure					
Second trimester	3	1.30	[1.03, 1.64]	0.027	0
Third trimester	3	1.16	[0.94, 1.42]	0.163	80.4
Adjusted factors					
Family history of allergy adjusted	9	1.17	[1.06, 1.30]	0.003	67.1
Eczema/atopic dermatitis assessment method					
Physician-diagnosed	4	1.25	[1.07, 1.45]	0.006	0
Parent-reported	6	1.08	[1.00, 1.16]	0.042	81.4

95% CI, 95% Confidence intervals; AD, Atopic dermatitis; No., Number; OR, Odds ratio; STAI, State Trait Anxiety Inventory.

### Publication bias and sensitivity analyses

After removing certain studies, the sensitivity analysis found no significant association between maternal anxiety and offspring eczema/AD (Supplementary Figure S1). Begg's test for this association revealed no significant publication bias (*P* = 0.858, Supplementary Figure S2). In contrast, the sensitivity analysis for maternal depression and offspring eczema/AD indicated that the pooled results were stable (Supplementary Figure S3). Similarly, Begg's test detected no significant publication bias for the association involving maternal depression (*P* = 0.721, Supplementary Figure S4).

## Discussion

This meta-analysis aggregated data from 12 high-quality cohort studies to assess the relationship between maternal anxiety/depression during pregnancy and the development of eczema/AD in offspring. Our results demonstrated that maternal anxiety and depression during pregnancy were potentially associated with a higher incidence eczema/AD in children when compared to control groups. The potential underlying mechanisms may include the following:

A study has identified a robust genetic correlation between AD and major depressive disorder (MDD), revealing 11 pleiotropic loci that are jointly associated with both conditions. Of these, six loci exhibited concordant effects in the AD-MDD pair. Notably, a significant pleiotropic region was found within the 5q31.1 cytokine gene cluster, which includes *interleukin (IL)-4 and IL-13*, both recognized as risk genes for AD. Additionally, gene-set analysis indicated substantial enrichment in inflammatory pathways, such as leukocyte differentiation, which are implicated in both AD and depression (23, 24). This genetic predisposition may be transmitted from mother to offspring which could facilitate T helper 2 (Th2) cell differentiation and may contribute to dysregulated immune responses in subsequent generations (25–27).

Another study suggested that maternal anxiety might influence the onset of AD in descendants by modifying placental DNA methylation. Anxiety is thought to elevate tumor necrosis factor-α levels, which, in turn, induce hypomethylation of a particular CpG site in the *matrix metalloproteinase (MMP) 27* gene, resulting in its upregulation (28, 29). MMP27, a member of the MMP family, is implicated in inflammation through the regulation of inflammatory mediators, establishment of chemokine gradients to facilitate inflammatory cell recruitment, and cleavage of endothelial junctional proteins to compromise barrier integrity (30). Consequently, MMP27 protein participation in inflammatory processes may contribute to the pathogenesis of AD. Additionally, genetic factors could also influence impaired skin barrier function. Evidence suggests that Western patients with AD exhibit a higher prevalence of filaggrin gene mutations than their Asian counterparts, resulting in impaired filaggrin function, compromising the skin barrier, and increasing the susceptibility to AD onset and aggravation (31, 32). This trend was consistent with findings from subgroup analysis on anxiety, showing a stronger association of maternal anxiety with offspring eczema/AD development in Western nations (OR = 1.34) than in Eastern regions (OR = 1.13). Conversely, the subgroup analysis on depression indicated a similar influence of maternal depression on offspring eczema and AD in both Eastern and Western countries, albeit the association in the Western subgroup lacked statistical significance. These outcomes could potentially be attributed to inadequate sample sizes.

As previously mentioned, the expression of the *IL-4 and IL-13* genes is upregulated. Elevated levels of these pro-inflammatory cytokines, specifically IL-4 and IL-13, have been observed in mothers experiencing depression or anxiety during pregnancy (29, 33–35). These cytokines may traverse the placental barrier and enter fetal circulation. Immunologically, studies have shown that IL-4 and IL-13 facilitate Th2 cell differentiation, IgE synthesis, and the activation of eosinophils and mast cells, thereby inciting inflammation and pruritus (36–39). Concerning skin barrier function, these cytokines downregulate essential proteins, including filaggrin, and impair lipid synthesis, disrupt keratinocyte differentiation, and reduce antimicrobial peptide production (40–42). Collectively, these effects may contribute to xerosis, compromised skin integrity, and heightened susceptibility to infection. Additionally, IL-13 contributes to dermal thickening and lichenification, which are characteristic of chronic AD, by stimulating collagen accumulation and fibrotic processes (43, 44). Beyond genetic factors, researchers have proposed that prenatal stress may contribute to elevated levels of IL-4 and IL-13 through mechanisms such as the activation of the hypothalamic-pituitary-adrenal (HPA) axis and a Th2-skewed immune response, which suggests a potential pathway that could be associated with an increased incidence of eczema/AD in offspring (45, 46). To investigate this multifactorial pathway, a subgroup analysis was conducted, adjusting for a family history of allergic diseases. The results indicated that offspring in the exposed group showed a higher incidence of developing eczema/AD compared to the control group. Taken together, these findings suggest that the development of eczema/AD in offspring involves environmental factors in addition to genetic predisposition.

Apart from the pro-inflammatory cytokine environment, the dysregulation of the maternal HPA axis may represent another crucial pathway linking maternal anxiety/depression during pregnancy to the development of eczema or AD in offspring. Depressed pregnant individuals have been found to exhibit increased levels of placental corticotropin-releasing hormone (pCRH) in their plasma, likely influenced by heightened cortisol levels, the final product of the HPA axis, which elevate in response to maternal anxiety and depression. pCRH is widely recognized as a significant factor in shaping fetal HPA axis development. This increase elevates fetal glucocorticoid levels, resulting in supraphysiological exposure of the fetus (47, 48). Studies on animals indicated that such prenatal exposure to glucocorticoids could alter the fetal HPA axis, resulting in functional disruptions (49). Patients with AD have shown abnormalities in the HPA axis, possibly indicating compromised HPA-mediated immune regulation, thereby increasing the vulnerability of offspring to AD and other inflammatory conditions (50). A recent study detailed a potential pathway from maternal stress to offspring eczema. Maternal prenatal stress similarly elevates maternal HPA-axis activity and systemic glucocorticoid levels, resulting in increased corticosterone concentrations in maternal plasma and amniotic fluid. The excess corticosterone penetrates the fetal environment and interacts with cutaneous mast cells, which express the glucocorticoid receptor abundantly. This interaction activates glucocorticoid receptor-mediated signaling, thereby reprogramming the developmental trajectory of these cells. Consequently, fetal mast cells adopt a transcriptional and functional profile that favors pro-inflammatory activity. Postnatally, these developmentally programmed mast cells exhibit exaggerated responses to otherwise innocuous mechanical stimuli, leading to aberrant cutaneous inflammation and the onset of neonatal eczema (51). It is important to note that the potential role of immune pathways in offspring eczema/AD likely originates during early fetal development. This mechanism is rooted in the fact that the programming and establishment of the fetal immune system are predominantly completed in the early stages of pregnancy and mature by the later stages (52–54). Our subgroup analysis further corroborated this mechanism, indicating a stronger association between maternal exposure to anxiety/depression during early pregnancy and offspring eczema/AD.

According to experimental animal research, airborne *Staphylococcus aureus (S. aureus)* may play a role in maternal depression through mechanisms involving abnormal neural oscillations and mitochondrial dysfunction (55). Similarly, newborns exposed to this environment may experience cutaneous colonization by S. aureus and disruption of their normal microbial flora. Studies indicate that *S. aureus* may also contribute to the development and exacerbation of AD through several, pathways, including the release of staphylococcal enterotoxins that activate T cells and induce AD-like lesions on the skin; alteration of Langerhans cell-mediated T cell responses, resulting in a Th2 bias; induction of IL-1*α* production, which amplifies cutaneous inflammation; and promotion of fibrinogen expression in the skin, thereby enhancing bacterial colonization (56, 57). Furthermore, the gut microbiome merits further investigation. Studies have shown an increased presence of *Klebsiella* in the gut microbiota of individuals suffering from depression and in children with AD, possibly due to the vertical transmission of gut microorganisms (58, 59). The higher levels of *Klebsiella* may hinder the colonization of beneficial bacteria such as *Bacteroides fragilis (B. fragilis)*, disrupting the normal gut microbial community structure (60). Studies have demonstrated that *B. fragilis* exhibits anti-inflammatory properties, which are mediated primarily through the production of a polysaccharide that has been shown to help correct systemic T cell defects and maintain a proper Th1/Th2 balance (61). Therefore, a decrease in *B. fragilis* abundance may thus reduce its protective role against eczema, possibly by limiting the production of its immunomodulatory polysaccharide (62). Ultimately, lipopolysaccharides from *K*lebsiella may activate the TLR4 receptor pathway, promoting the release of pro-inflammatory cytokines, worsening immune dysregulation, and allergic responses, potentially serving as an underlying cause of AD (60, 63).

Such psychological stress often does not exist in isolation but may interact with various factors such as environmental conditions and air pollution to influence offspring health. Notably, a recent study provides further insight into the association between parental stress and offspring eczema. The study indicated that this association is mediated through a stress-environment interaction (64). Specifically, while psychosocial stress increased eczema prevalence, socioeconomic stress further amplified the effects of exposure indoor environmental factors and outdoor air pollution on the disease. These findings offer important support and extension to our study. First, this may help explain the substantial heterogeneity across studies, given the varying levels of environmental exposure and types of stress in different populations. Second, the research indicates that maternal depression and anxiety likely correlate with psychosocial stress, socioeconomic stress, and environmental exposures, introducing potential confounding bias.

Eczema refers to a skin condition with various etiologies, often influenced by factors such as friction, chemical stimuli, and sweating (65, 66). Its broad diagnostic criteria may further obscure the association with maternal depression and anxiety. In contrast, AD is a well-defined inflammatory skin condition characterized by specific pathological mechanisms, including S. aureus colonization and genetic factors, which directly initiate and promote inflammatory processes, thereby allowing for a stronger causal inference (57, 67, 68). Supporting this mechanistic distinction, our subgroup analysis indicated that maternal anxiety or depression during pregnancy was significantly associated with offspring AD compared to the control group. However, no significant difference in the prevalence of eczema was observed between these groups.

No significant differences in the prevalence of offspring eczema/AD were observed in the group where maternal anxiety during pregnancy was evaluated using either STAI-S or STAI-T, in comparison to the control group. The primary factor accounting for this lack of differentiation is primarily the insufficient sample size. Furthermore, stricter diagnostic criteria by physicians and potential parental reporting bias led to an association between physician-diagnosed eczema/AD and maternal depression/anxiety in subgroup analysis, while no significant association was evident in the parent-reported subgroup. This disparity implies that the absence of a significant connection in the parent-reported subgroup may stem from under-diagnosis or a stronger impact of confounding variables (69).

The administration of antidepressants or anxiolytics during pregnancy significantly impacts offspring development. Ideally, comparisons should be drawn between medicated and non-medicated groups of mothers experiencing anxiety or depression during pregnancy, accompanied by relevant subgroup analyses. However, due to the fact that not all mothers with anxiety or depression received medication and the included studies lacked pertinent data, such subgroup analyses were not feasible. Furthermore, the potential influence of antidepressants and anxiolytics themselves on the development of eczema and dermatitis in offspring should also be considered, representing a significant limitation of our study. Future research in this area should utilize well-designed observational studies and animal experiments to elucidate the potential relationship. Regarding outcome assessment, the measurement of eczema/AD outcomes presents methodological limitations. The reliance on parental reporting or physician diagnosis in the included studies may introduce information bias, a limitation that should be acknowledged. In addition, our analysis was limited to assessing exposures within specific trimesters and did not evaluate the cumulative effect across the entire pregnancy. Specifically, the available data are insufficient to investigate whether the total duration of maternal anxiety/depression or exposure during a particular trimester is more strongly associated with the development of eczema/AD in offspring. This gap in our analysis constitutes another significant limitation. Given the limited overall sample size and the paucity of studies within individual subgroups, a reliable meta-regression analysis for factors like region, outcome definition, or assessment tools was not feasible, which represents one of the limitations of this study. Additionally, the observed association for maternal anxiety was not stable in sensitivity analyses, calling for a cautious interpretation of this exploratory finding.

Our study surpasses prior meta-analyses in several aspects. By including cohort studies, we bolstered the quality of evidence. Additionally, integrating recently published research enhances result reliability. Furthermore, we performed subgroup analyses considering crucial factors like disease types, region, and timing of exposure to identify factors that may influence the observed association. This research underscores the significance of vigilantly monitoring and promoting maternal psychological health during pregnancy. Prompt interventions to mitigate maternal depression and anxiety may positively impact offspring skin health.

## Conclusion

This meta-analysis indicates a potential association between maternal anxiety/depression during pregnancy and the development of eczema/AD in offspring. Furthermore, disease types, region, timing of exposure, eczema/AD assessment method may influence this association.

## Data Availability

The original contributions presented in the study are included in the article/Supplementary Material, further inquiries can be directed to the corresponding author/s.

## Supplementary material

Supplementary Figure S1 Sensitivity analyses of the maternal anxiety and the development of offspring eczema/AD in offspring.

Supplementary Figure S2 Begg's text of the meta-analysis on maternal anxiety and the development of offspring eczema/AD in offspring (p = 0.858).

SUpplementary Figure S3 Sensitivity analyses of the maternal depression and the development of offspring eczema/AD in offspring.

Supplementary Figure S4 Begg's text of the meta-analysis on maternal depression and the development of offspring eczema/AD in offspring (p = 0.721).

Supplementary Table S1 Characteristics of included studies in the meta-analysis.

Supplementary Table S2 Quality assessment of cohort studies included.
