# Supplementary figures and images for: Association between maternal anxiety/depression in pregnancy and the development of offspring eczema/AD: a meta-analysis based on cohort studies
Source: Front Pediatr. 2026 Jan 13;13:1734662. doi: 10.3389/fped.2025.1734662 (PMC12835386; doi:10.3389/fped.2025.1734662)

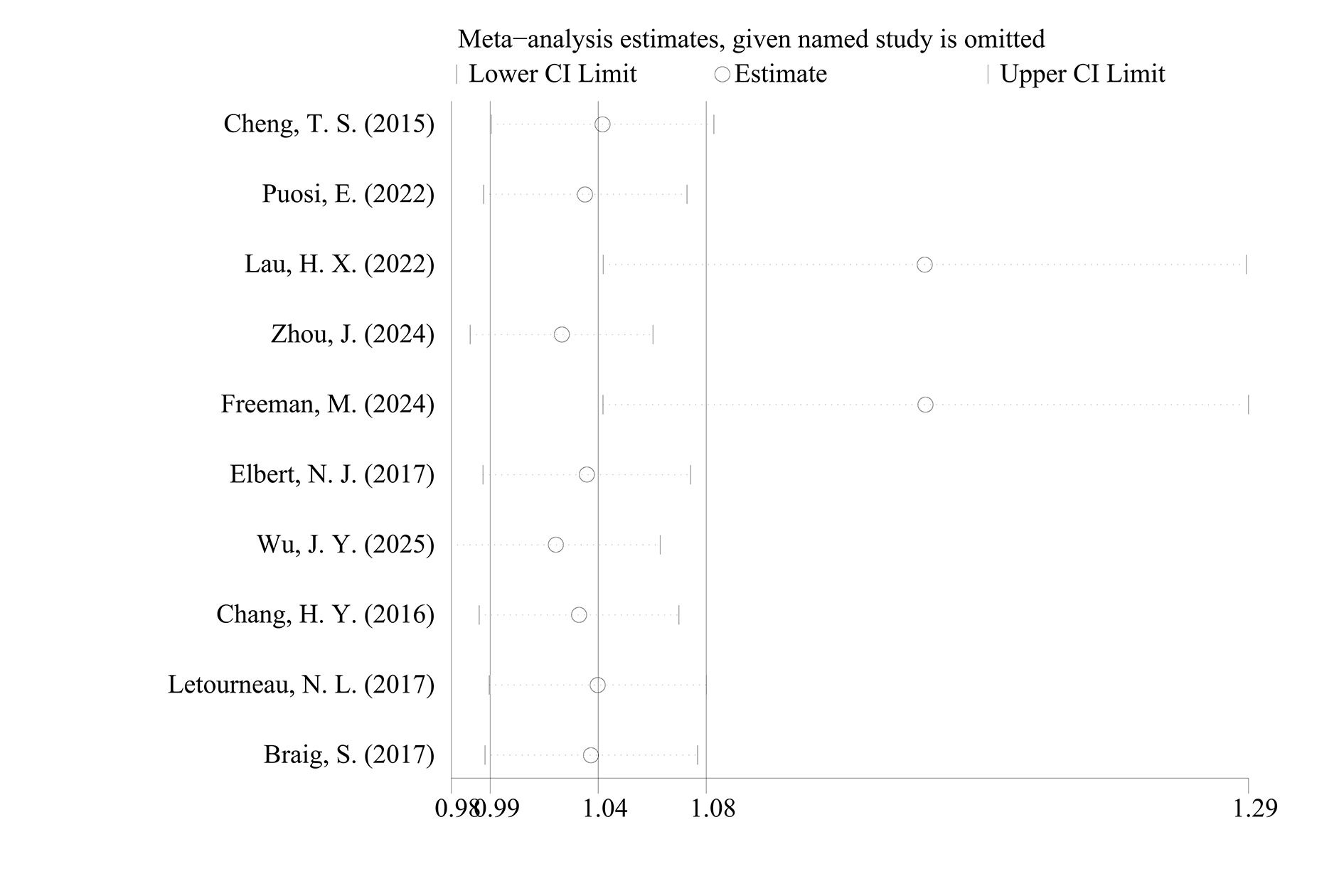

Supplement: Supplementary Figure S1 — Sensitivity analyses of the maternal anxiety and the development of offspring eczema/AD in offspring. [file Image1.tif]

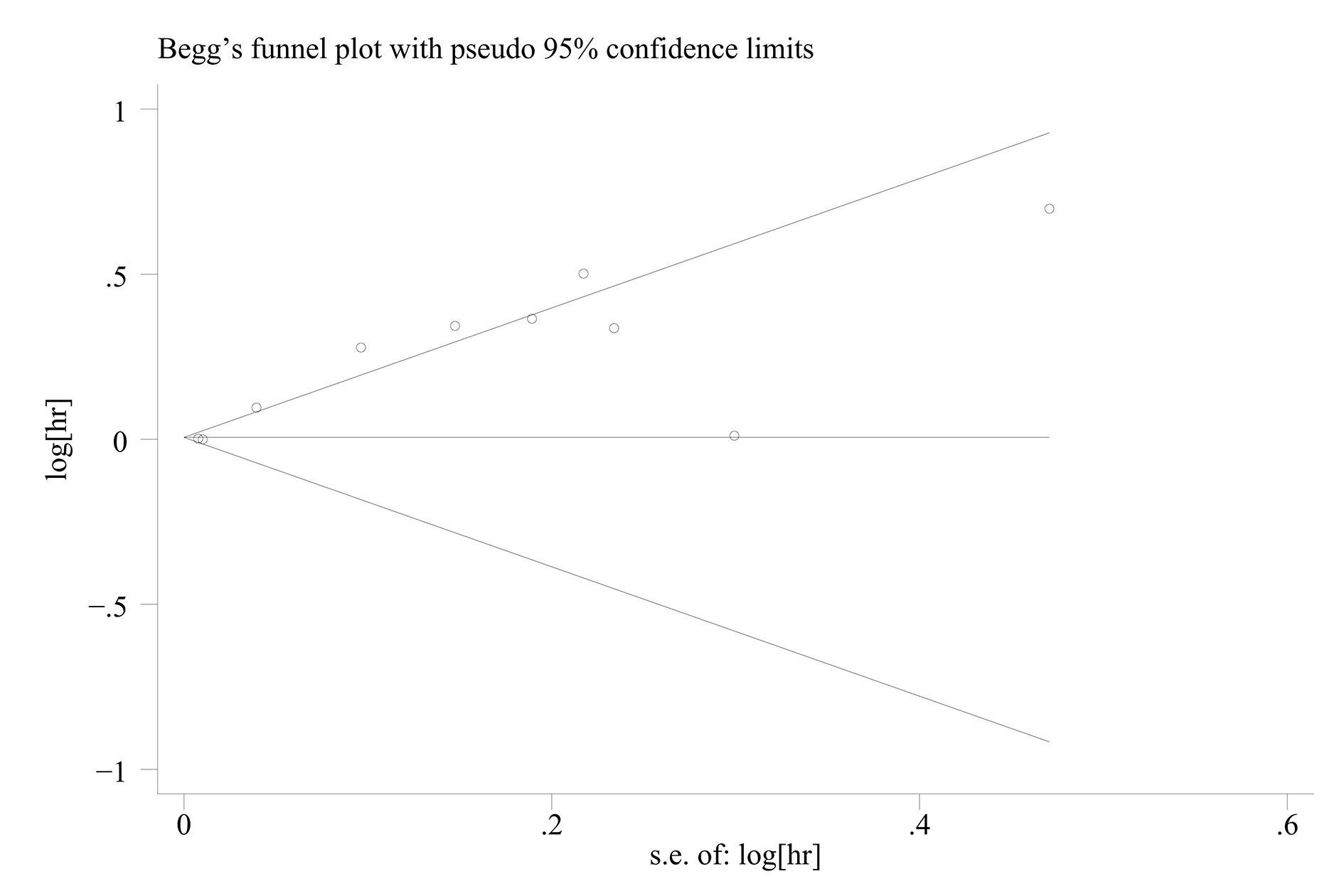

Supplement: Supplementary Figure S2 — Begg's text of the meta-analysis on maternal anxiety and the development of offspring eczema/AD in offspring (p = 0.858). [file Image2.tif]

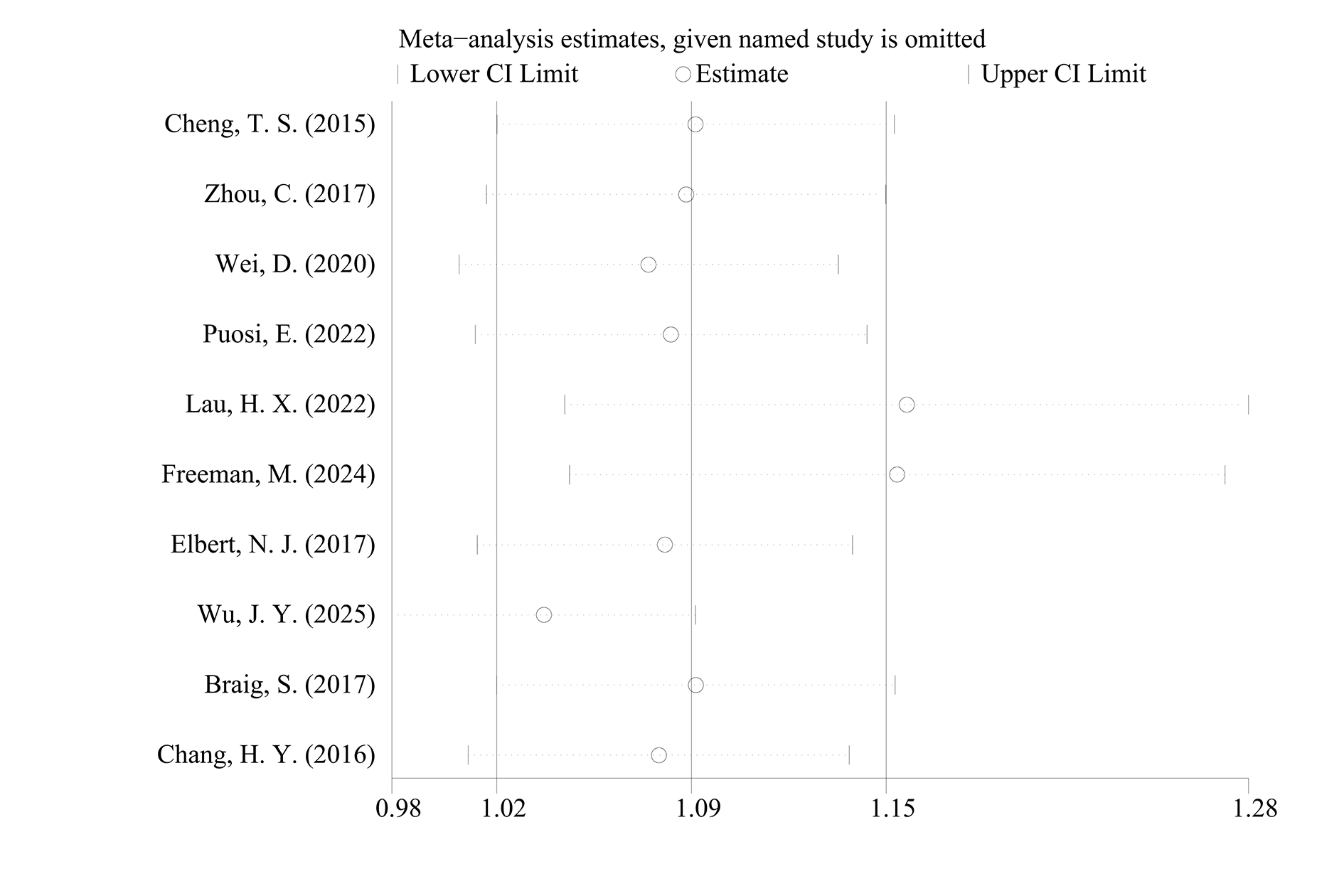

Supplement: SUpplementary Figure S3 — Sensitivity analyses of the maternal depression and the development of offspring eczema/AD in offspring. [file Image3.tif]

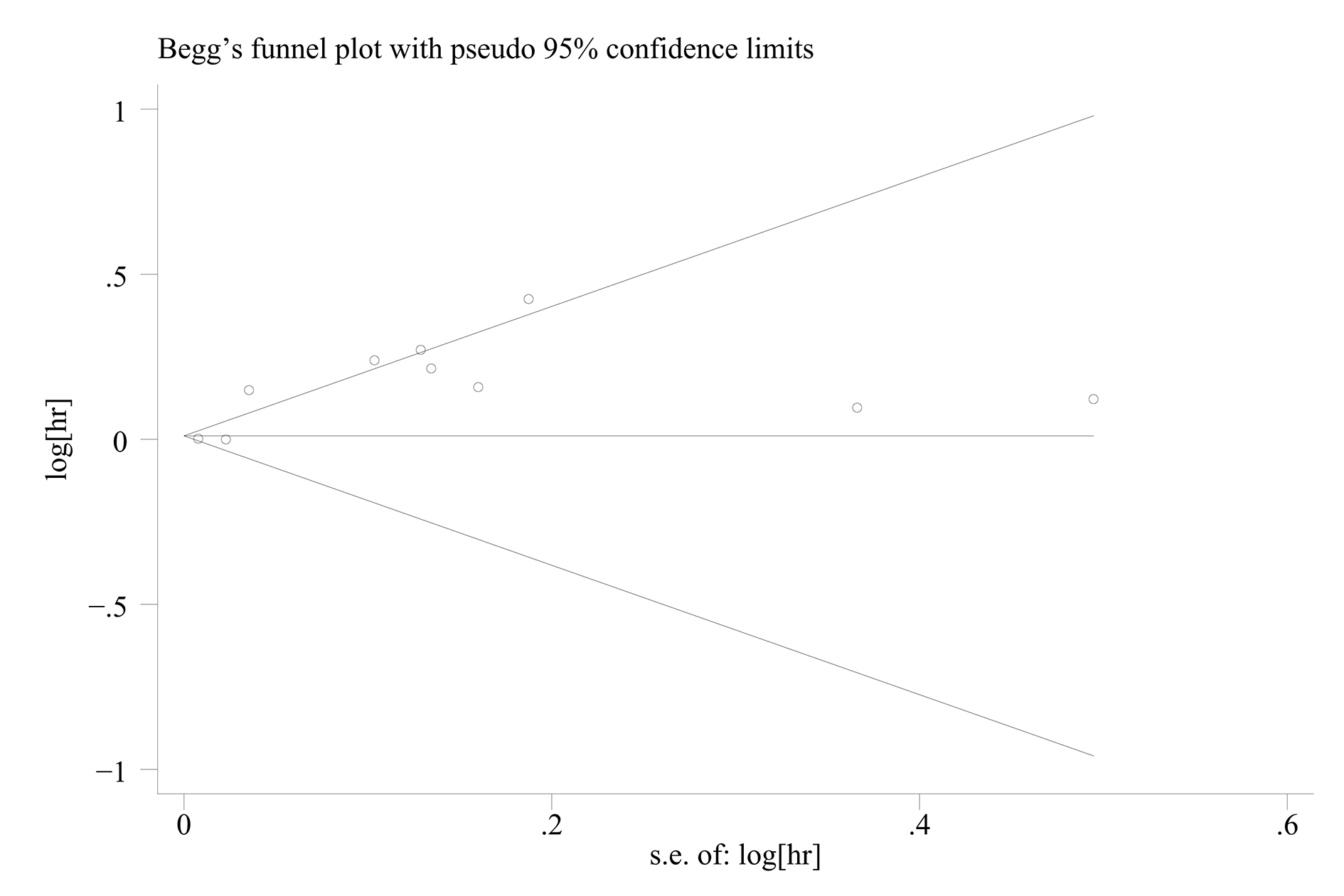

Supplement: Supplementary Figure S4 — Begg's text of the meta-analysis on maternal depression and the development of offspring eczema/AD in offspring (p = 0.721). [file Image4.tif]
