# Supplementary material for: Association between maternal anxiety/depression in pregnancy and the development of offspring eczema/AD: a meta-analysis based on cohort studies
Source: Front Pediatr. 2026 Jan 13;13:1734662. doi: 10.3389/fped.2025.1734662 (PMC12835386; doi:10.3389/fped.2025.1734662)
Supplement: Supplementary Table S1 — Characteristics of included studies in the meta-analysis. [file Table1.docx]

Supplementary Table 1. Characteristics of included cohort studies in the meta-analysis.

| Author, year | Exposure window | Outcome Assessment Methods | Adjusted factors |
| --- | --- | --- | --- |
| Cheng, T. S. 2015 | 26 weeks of gestation | physician diagnosis | child’s sex, birthweight, gestational age, ethnicity, maternal age, maternal BMI, education, marital status, household income, maternal eczema history, paternal eczema history, maternal asthma history, maternal pre-pregnancy smoking and passive smoking during pregnancy |
| Zhou, C. 2017 | between 24 and 28 weeks of gestation | ISAAC questionnaires filled out by the parents | potential confounders of the study center, education, maternal smoking during pregnancy, maternal age, maternal pre-pregnancy body mass index, siblings, child’s sex, and family history of asthma, eczema, allergic rhinitis, or food allergy |
| Wei, D. 2020 | <20th week of gestation  ≥33rd week of gestation to delivery | parental report of physician-diagnosed eczema | maternal age, maternal income, education, maternal active smoking during pregnancy, maternal passive smoking during pregnancy, pre-pregnancy BMI, parental history of allergic diseases, parity, and pregnancy complications |
| Puosi, E. 2022 | 14, 24, 34 weeks of gestation | doctor‐diagnosed eczema at the child according to the standardized questionnaire of ISAAC | child’s sex, maternal history of atopic diseases, and education |
| Lau, H. X. 2022 | at each trimester during pregnancy | maternally reported doctor diagnosis of eczema | period of maximum stress, ethnicity, maternal age, education, parity, smoking during pregnancy, maternal history of allergy, child’s sex and gestational age |
| Zhou, J. 2024 | during the first, second, and third trimesters of pregnancy | maternally reported doctor diagnosis of eczema and regarding children's eczema date by using standardized ISAAC questionnaires | maternal age, parity, education, pre-pregnancy BMI, residence, household income, maternal metabolic dysfunctions, smoking during pregnancy, alcohol use during pregnancy |
| Freeman, M. 2024 | during late pregnancy | participants report | ethnicity, education, household income, parity, gestational age, and child’s sex |
| Elbert, N. J. 2017 | in the second trimester of pregnancy | parental reported physician-diagnosed eczema | maternal age, education, ethnicity, history of allergy, eczema or asthma, parity, pet keeping, body mass index at enrollment, smoking, and child’s sex, gestational age and birth weight |
| Wu, J. Y. 2025 | before 14 gestational weeks | assessed through questionnaires about maternal report of physician diagnosis | maternal age, education, maternal pregnancy BMI, household income, birth season, child’s sex and maternal history of asthma, atopic dermatitis or allergic rhinitis |
| Chang, H. Y. 2016 | 36 weeks of gestation | physician diagnosis | maternal age, education, delivery method, birth season, maternal history of allergic diseases, and child’s sex |
| Letourneau, N. L. 2017 | NA | mothers reported physician diagnosis of infant atopic dermatitis | maternal unresponsiveness and controlling, postnatal depression and anxiety, pregnancy specific anxiety, maternal asthma |
| Braig, S. 2017 | NA | Self-administered parental questionnaires | child’s sex, gestational age, maternal and paternal atopy, maternal smoking during pregnancy, maternal BMI, maternal age, birth weight |

NA: not available; BMI: Body Mass Index; ISAAC: International Study of Asthma and Allergies in Childhood.
