# Supplementary material for: Association between maternal anxiety/depression in pregnancy and the development of offspring eczema/AD: a meta-analysis based on cohort studies
Source: Front Pediatr. 2026 Jan 13;13:1734662. doi: 10.3389/fped.2025.1734662 (PMC12835386; doi:10.3389/fped.2025.1734662)
Supplement: Supplementary Table S2 — Quality assessment of cohort studies included. [file Table2.docx]

**Supplementary Table 2. Quality assessment of cohort studies included.**

| Author, year | **Selection (Out of 4)** | | | | **Comparability**  **(Out of 2)** | **Outcomes (Out of 3)** | | | **Total**  **(Out of 9)** |
| --- | --- | --- | --- | --- | --- | --- | --- | --- | --- |
|  | Representativeness of exposed cohort | Selection of non exposed cohort | Ascertainment  of exposure | Outcome not present at the start of the study |  | Assessment of outcomes | Length of follow-up | Adequacy of follow up of cohorts |  |
| Cheng, T. S. 2015 | 1 | 1 | 1 | 1 | 2 | 1 | 0 | 1 | 8 |
| Zhou, C. 2017 | 1 | 1 | 1 | 1 | 1 | 1 | 1 | 1 | 8 |
| Wei, D. 2020 | 1 | 1 | 1 | 1 | 1 | 1 | 0 | 1 | 7 |
| Puosi, E. 2022 | 1 | 1 | 1 | 1 | 1 | 1 | 0 | 1 | 7 |
| Lau, H. X. 2022 | 1 | 1 | 1 | 1 | 2 | 1 | 0 | 1 | 8 |
| Zhou, J. 2024 | 1 | 1 | 1 | 1 | 1 | 1 | 1 | 1 | 8 |
| Freeman, M. 2024 | 1 | 1 | 1 | 1 | 1 | 0 | 1 | 1 | 7 |
| Elbert, N. J. 2017 | 1 | 1 | 1 | 1 | 1 | 1 | 1 | 1 | 8 |
| Wu, J. Y. 2025 | 1 | 1 | 1 | 1 | 2 | 0 | 0 | 1 | 7 |
| Chang, H. Y. 2016 | 1 | 1 | 1 | 1 | 1 | 1 | 0 | 1 | 7 |
| Letourneau, N. L. 2017 | 1 | 1 | 1 | 1 | 1 | 1 | 1 | 1 | 8 |
| Braig, S. 2017 | 1 | 1 | 1 | 1 | 2 | 0 | 1 | 1 | 8 |

The cohort studies were assessed by the Newcastle-Ottawa Quality Assessment Scale (NOS) checklist.
