# Supplementary material for: Association between maternal anxiety/depression in pregnancy and the development of offspring eczema/AD: a meta-analysis based on cohort studies
Source: Front Pediatr. 2026 Jan 13;13:1734662. doi: 10.3389/fped.2025.1734662 (PMC12835386; doi:10.3389/fped.2025.1734662)
Supplement: Supplementary file 7 [file Table3.docx]

**Supplementary** **Search Strategy.**

**Pubmed**

| Search number | Query | Search Details | Results |
| --- | --- | --- | --- |
| 1 | offspring | (“offspring”[All Fields] OR “newborn”[All Fields] OR “neonate”[All Fields] OR “infant”[All Fields] OR “baby”[All Fields] OR “progeny”[All Fields] OR “children”[All Fields] OR “child”[All Fields] OR “kids”[All Fields] OR “toddler”[All Fields] OR “adolescent”[All Fields] OR “teenager”[All Fields]) | 5467446 |
| 2 | atopic dermatitis OR eczema | (“Dermatitis, Atopic”[MeSH Terms] OR “atopic dermatitis”[All Fields] OR “dermatitis, atopic”[All Fields] OR “Dermatitis, Atopic”[All Fields] OR “eczema”[MeSH Terms] OR “eczema”[All Fields] OR “Eczematous Dermatitis”[All Fields]) | 61162 |
| 3 | maternal | (“pregnant”[All Fields] OR “pregnancy”[All Fields] OR “pregnant woman”[All Fields] OR “antenatal”[All Fields] OR “prenatal”[All Fields] OR “postpartum”[All Fields] OR “gestation”[All Fields] OR “perinatal”[All Fields] OR “pregnancy-related”[All Fields] OR “maternal”[All Fields]) | 1505376 |
| 4 | depression OR anxiety | (“depressive”[All Fields] OR “depression”[All Fields] OR “anxious”[All Fields] OR “anxiety”[All Fields] OR “depressive state”[All Fields]) | 846822 |
| 5 | (offspring) AND (atopic dermatitis OR eczema) AND (maternal) AND (depression OR anxiety) | (“offspring”[All Fields] OR “newborn”[All Fields] OR “neonate”[All Fields] OR “infant”[All Fields] OR “baby”[All Fields] OR “progeny”[All Fields] OR “children”[All Fields] OR “child”[All Fields] OR “kids”[All Fields] OR “toddler”[All Fields] OR “adolescent”[All Fields] OR “teenager”[All Fields]) AND (“Dermatitis, Atopic”[MeSH Terms] OR “atopic dermatitis”[All Fields] OR “dermatitis, atopic”[All Fields] OR “Dermatitis, Atopic”[All Fields] OR “eczema”[MeSH Terms] OR “eczema”[All Fields] OR “Eczematous Dermatitis”[All Fields]) AND (“pregnant”[All Fields] OR “pregnancy”[All Fields] OR “pregnant woman”[All Fields] OR “antenatal”[All Fields] OR “prenatal”[All Fields] OR “postpartum”[All Fields] OR “gestation”[All Fields] OR “perinatal”[All Fields] OR “pregnancy-related”[All Fields] OR “maternal”[All Fields]) AND (“depressive”[All Fields] OR “depression”[All Fields] OR “anxious”[All Fields] OR “anxiety”[All Fields] OR “depressive state”[All Fields]) | 154 |

**Web of Science**

| Search number |  | Search Details | Results |
| --- | --- | --- | --- |
| 1 | TS = (offspring) | TS = (“offspring” OR “newborn” OR “neonate” OR “infant” OR “baby” OR “progeny” OR “children” OR “child” OR “kids” OR “toddler” OR “adolescent” OR “teenager”) | 1433487 |
| 2 | TS = (atopic dermatitis OR eczema) | TS = (“Dermatitis, Atopic” OR “atopic dermatitis” OR “eczema” OR “Eczematous Dermatitis”) | 46659 |
| 3 | TS = (maternal) | TS = (“pregnant” OR “pregnancy” OR “pregnant woman” OR “antenatal” OR “prenatal” OR “postpartum” OR “gestation” OR “perinatal” OR “pregnancy-related” OR “maternal”) | 621925 |
| 4 | TS = (depression OR anxiety) | TS = (“depressive” OR “depression” OR “anxious” OR “anxiety” OR “depressive state”) | 582546 |
| 5 | (TS= (offspring)) AND (TS = (atopic dermatitis OR eczema)) AND (TS = (maternal)) AND (TS = (depression OR anxiety)) | (TS = (“offspring” OR “newborn” OR “neonate” OR “infant” OR “baby” OR “progeny” OR “children” OR “child” OR “kids” OR “toddler” OR “adolescent” OR “teenager”)) AND (TS = (“Dermatitis, Atopic” OR “atopic dermatitis” OR “eczema” OR “Eczematous Dermatitis”)) AND (TS = (“pregnant” OR “pregnancy” OR “pregnant woman” OR “antenatal” OR “prenatal” OR “postpartum” OR “gestation” OR “perinatal” OR “pregnancy-related” OR “maternal”)) AND (TS = (“depressive” OR “depression” OR “anxious” OR “anxiety” OR “depressive state”)) | 148 |

**Embase**

| Search number | Query | Search Details | Results |
| --- | --- | --- | --- |
| 1 | offspring | (offspring OR newborn OR neonate OR infant OR baby OR progeny OR children OR child OR kids OR toddler OR adolescent OR teenager).mp. | 5277824 |
| 2 | atopic dermatitis OR eczema | (Dermatitis, Atopic OR atopic dermatitis OR eczema OR Eczematous Dermatitis).mp. | 115009 |
| 3 | maternal | (pregnant OR pregnancy OR pregnant woman OR antenatal OR prenatal OR postpartum OR gestation OR perinatal OR pregnancy-related OR maternal).mp. | 1695696 |
| 4 | depression OR anxiety | (depressive OR depression OR anxious OR anxiety OR depressive state).mp. | 11360333 |
| 5 | #1 AND #2 AND #3 AND #4 | #1 AND #2 AND #3 AND #4 | 317 |

**Cochrane**

| Search number | Query | Search Details | Results |
| --- | --- | --- | --- |
| 1 | offspring | (offspring) OR (newborn) OR (neonate) OR (infant) OR (baby) OR (progeny) OR (children) OR (child) OR (kids) OR (toddler) OR (adolescent) OR (teenager) | 367308 |
| 2 | atopic dermatitis | (Dermatitis, Atopic) OR (atopic dermatitis) | 7258 |
| 3 | eczema | (eczema) OR (Eczematous Dermatitis) | 5993 |
| 4 | maternal | (pregnant) OR (pregnancy) OR (pregnant woman) OR (antenatal) OR (prenatal) OR (postpartum) OR (gestation) OR (perinatal) OR (pregnancy-related) OR (maternal) | 119530 |
| 5 | depression | (depressive) OR (depression) OR (depressive state) | 127525 |
| 6 | anxiety | (anxious) OR (anxiety) | 95193 |
| 4 | #1 AND (#2 OR #3) AND #4 AND (#5 OR #6) | #1 AND (#2 OR #3) AND #4 AND (#5 OR #6) | 79 |

Based on the aforementioned search strategy, searches were conducted in the above four electronic databases from the inception of each database until July 2025. A total of 154 articles were retrieved from PubMed, 148 from Web of Science, 317 from Embase, and 79 from the Cochrane Library, resulting in an initial pool of 698 articles. To ensure comprehensiveness, the references of all included articles were also screened to identify additional eligible studies; however, no further eligible articles were obtained through this method. After removing duplicates, 496 unique articles remained. Two reviewers independently evaluated these records based on titles and abstracts, excluding 462 irrelevant articles and retaining 34 for full-text assessment. Following the exclusion of seven review articles, one case-control study, two non-English publications, three articles with unavailable data, and nine articles that did not report relevant outcomes, 12 studies met the criteria for meta-analytic synthesis and were included in the meta-analysis.
